# Mutations in the PKM2 exon-10 region are associated with reduced allostery and increased nuclear translocation

**DOI:** 10.1038/s42003-019-0343-4

**Published:** 2019-03-15

**Authors:** Tsan-Jan Chen, Hung-Jung Wang, Jai-Shin Liu, Hsin-Hung Cheng, Sheng-Chieh Hsu, Meng-Chen Wu, Chien-Hung Lu, Yu-Fang Wu, Jing-Wen Wu, Ying-Yuan Liu, Hsing-Jien Kung, Wen-Ching Wang

**Affiliations:** 1 Institute of Molecular and Cellular Biology and Department of Life Science, National Tsing-Hua University, Hsinchu, 30013 Taiwan; 2 Institute of Biotechnology and Pharmaceutical Research, National Health Research Institutes, Miaoli, 35053 Taiwan; 3 Institute of Biotechnology, National Tsing-Hua University, Hsinchu, 30013 Taiwan; 4 Institute of Molecular and Genomic Medicine, National Health Research Institutes, Miaoli, 35053 Taiwan; 5 Department of Biochemistry and Molecular Medicine, University of California Davis School of Medicine, University of California Davis Cancer Centre, Sacramento, CA 95817 USA

## Abstract

PKM2 is a key metabolic enzyme central to glucose metabolism and energy expenditure. Multiple stimuli regulate PKM2’s activity through allosteric modulation and post-translational modifications. Furthermore, PKM2 can partner with KDM8, an oncogenic demethylase and enter the nucleus to serve as a HIF1α co-activator. Yet, the mechanistic basis of the exon-10 region in allosteric regulation and nuclear translocation remains unclear. Here, we determined the crystal structures and kinetic coupling constants of exon-10 tumor-related mutants (H391Y and R399E), showing altered structural plasticity and reduced allostery. Immunoprecipitation analysis revealed increased interaction with KDM8 for H391Y, R399E, and G415R. We also found a higher degree of HIF1α-mediated transactivation activity, particularly in the presence of KDM8. Furthermore, overexpression of PKM2 mutants significantly elevated cell growth and migration. Together, PKM2 exon-10 mutations lead to structure-allostery alterations and increased nuclear functions mediated by KDM8 in breast cancer cells. Targeting the PKM2-KDM8 complex may provide a potential therapeutic intervention.

## Abstract (web summary)

Tsan-Jan Chen, Hung-Jung Wang et al. report the crystal structures of tumor-related mutant forms of pyruvate kinase M2 (PKM2). They show that these mutations lead to increased interaction with the PKM2 binding partner KDM8, suggesting a mechanism for their oncogenic action.

## Introduction

Pyruvate kinase is the last-step enzyme in glycolysis that catalyzes the conversion of phosphoenolpyruvate to pyruvate while phosphorylation of ADP to produce ATP^1^. There are four isoforms (L, R, M1, and M2) of pyruvate kinase in mammals. The L and the R isoforms are encoded by *PKLR*, while M1 and M2 isoforms are encoded by *PKM*, differing by a single exon through the mutually exclusive alternative splicing of a pair of exons (exons 9 and 10) to produce PKM1 or PKM2 (for review see ref. ^2,3^). PKM2 is universally expressed in embryos^4,5^. In adults, PKM2 is often expressed in adipose tissue and pancreatic islets and PKM1 in muscle and brain, while PKL is mainly expressed in liver, kidney, and small intestine and PKR in erythrocytes. Interestingly, PKM2 is also preferentially expressed in regenerating liver, and in cancer^4,5^. Elevated expression of PKM2 is observed in the cancerous tissues of essentially all cancer types using the cancer genome atlas (TCGA) datasets^6^, suggesting the selection of PKM2 expression favored by cancer cells.

PKM1 and PKM2 share an overall homologous fold but differ in a 45-amino acid stretch in the C-terminal domain of PKM2 (389‒433 residues of PKM2)^7^. PKM1 is constitutively stable and active as a tetrameric form, whereas PKM2 is allosterically controllable and can exist as a tetramer, dimer, or monomer in response to various effectors. The introduction of a well-known allosteric activator, fructose 1,6-bisphosphate (FBP), for instance, shifts a less active T-state into a fully active R-state tetrameric form^7^. Recently, metabolites, including serine (Ser), phenylalanine (Phe), succinylaminoimidazolecarboxamide ribose-5′-phosphate (SAICAR), and 3,3′,5-triiodothyroxine (T3), are being identified as allosteric effectors of PKM2^8–10^. Notably, the abundance of the dimeric PKM2 in tumor cells is correlated with increased glucose uptake and lactate production, referred to as aerobic glycolysis or Warburg metabolism^1,11,12^. Switching from PKM2 to PKM1 reverses the Warubrug metablolism in different cancer lines and reduces the tumor growth in nude mice^13^. Furthermore, potent small-molecule activators that stabilize the tetrameric form of PKM2 interfere with anabolic metabolism and suppress tumor growth^14^.

PKM2, mostly in its dimeric/monomeric state, is capable of translocating into the nucleus via interaction with importin α5^15^, wherein it serves as transcriptional coactivator of HIF1α and β-catenin^16,17^ or as a protein kinase phosphorylating nuclear proteins histone H3 and Stat3, further facilitating cell proliferation^15,18–20^. At the same time, the cytosolic pyruvate kinase activity is reduced, leading to the accumulation of glycolysis intermediates, fueling the biosynthesis of nucleotides, amino acids and lipids; this also additionally reduces the pyruvate kinase activity of PKM2. We have previously shown that PKM2 makes partnership with the oncogenic histone demethylase KDM8, which also promotes PKM2 nuclear translocation^21^. KDM8, a crucial factor for embryogenesis^22^, oncogenesis^22,23^, and stem-cell renewal^24^, is overexpressed and amplified in various tumor tissues^23,25,26^. It regulates the cell cycle by upregulating the expression of cyclin A^23,27,28^, and down-modulating that of p53 and p21^22^. Because KDM8 is expressed in virtually all tumor cells^21^, its role as a nuclear translocator of PKM2 in the context of oncogenesis deserves some attention.

The tetramer-dimer-monomer ratio of PKM2 is also regulated by post-translational modifications (PTMs). For instance, Lys433 of PKM2 is acetylated in the presence of mitogenic or oncogenic stimuli, Tyr105 is phosphorylated by growth factor signals, and Lys305 is acetylated by high glucose, shifting to the less active dimeric form of the enzyme^29–32^. However, the mechanistic basis of the exon-10 region in structure flexibility, allosteric regulation and the nuclear translocation in the context of KDM8 remains to be elucidated.

In this investigation, three tumor-related mutants, H391Y, R399E, and G415R^18,33–35^, were chosen to characterize the allostery and nuclear translocation activity and its relationship with KDM8. H391Y, a variant from a Bloom syndrome patient, exhibits a cross-monomer interaction with wild-type PKM2 for affecting PKM2 oligomerization and cell growth^34^. Bloom syndrome patients exhibiting BLM deficiency are characterized by a significantly increased incidence of all types of cancer at an early age^36^. R399E, an engineered, oncogenic mutant at the C–C interface region, substantially promoted tumor growth in a nude mouse model^18^. G415R, whose residue is situated at the dimer–dimer interface, was identified from a patient with kidney cancer^35^. Here, we demonstrated that these variants displayed considerably reduced allosteric regulation. We determined the crystal structures of H391Y and R399E, revealing mutation-driven conformational changes at the C–C interface, which are responsible for the substantial forfeiture of the allosteric conformational switch. In addition, compared with the wild-type enzyme, R399E, H391Y, and G415R mutants favorably interacted with KDM8 and exhibited enhanced nuclear translocation and HIF1α-mediated transactivation activity. These effects are even more pronounced in the presence of KDM8. Finally, we demonstrated that PKM2 mutants dramatically promoted tumor cell growth and migration.

## Results

### Clinical relevance of PKM2 and KDM8 in tumors

**Fig. 1:**
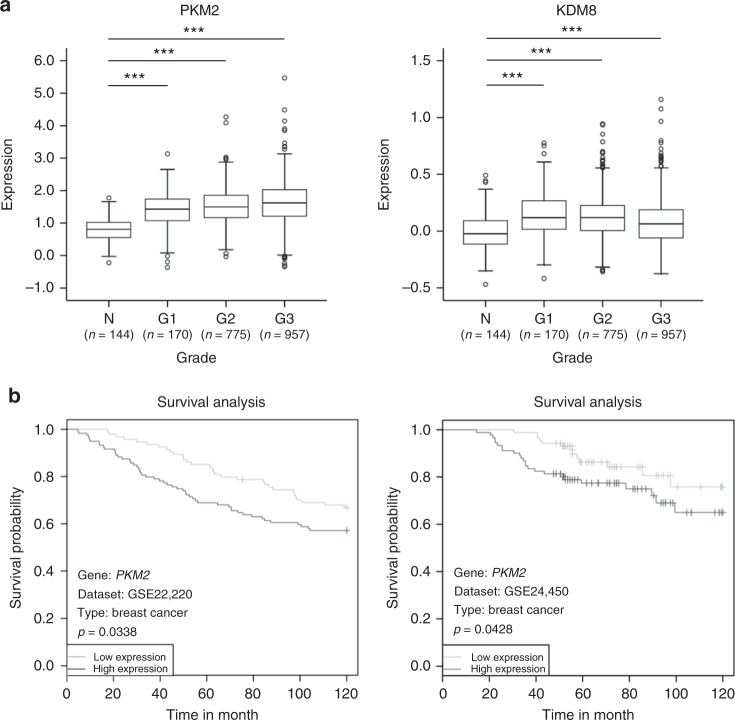
Clinical relevance of PKM2 and KDM8 in cancers. **a** PKM2 and KDM8 were overexpressed using METABRIC (the Molecular Taxonomy of Breast Cancer International Consortium) database (***p < 0.0001, two-tailed *t*-test). The source data is available in Supplementary Data 1. **b** Negative survival outcomes in two data sets from Table 1. N, normal. G1 to G3, grade 1 to grade 3

Data for immunohistochemical (IHC) staining from The Human Protein Atlas project (http://www.proteinatlas.org/) revealed that a majority of tumor tissues overexpress PKM2. In parallel, PKM2 was frequently overexpressed in tumor sections (the Oncomine^TM^ database; http://www.oncomine.org/), thus indicating its positive contribution to tumor growth. In addition, data from The Human Protein Atlas revealed that most malignant tumors also exhibited KDM8-positive IHC signals. Hsia et al. reported that KDM8 was overexpressed in multiple types of tumors, including breast, uterine, and liver tumors^23^. Notably, results of an analysis of a large collection of breast cancer tissues (*n* = 2509) from the Molecular Taxonomy of Breast Cancer International Consortium (METABRIC) database revealed that both PKM2 and KDM8 are significantly overexpressed in breast cancer samples compared with the normal control samples (*p* < 0.001)^37^ (Fig. 1a). Furthermore, mutual exclusivity and co-occurrence analyses of the METABRIC data set with mRNA expression *z*-scores ± 2.0 revealed that the co-occurrence of both PKM2 and KDM8 was statistically significant (*p* = 0.015)^25,26^. Similar results were also obtained for The Cancer Genome Atlas (TCGA, http://www.cbioportal.org/) kidney renal clear cell carcinoma (*n* = 538, *p* = 0.033). These results suggest co-occurring differential expression of PKM2 and KDM8 in these cancers.

We next utilized an interactome-based tool PRISURV to characterize the association of PKM2 and KDM8 expression levels with clinical outcomes^38^. PRISURV analysis revealed a large number of PKM2 interaction partners (*n* = 1081) but no known partners for KDM8. Further characterization indicated that the probed PKM2 interactomes were significantly associated with clinical outcomes in 12 clinical cancer data sets (Fig. 1b; Table 1). Of these, 11 had negative survival outcomes, including lung cancer (4), breast cancer (2), liposarcoma (1), prostate cancer (1), leukemia (1), colon cancer (1), and glioma (1), which is consistent with the positive contribution of PKM2 to cancer metabolism.

**Table 1:** PRISURV analysis of PKM2 interactomes

GEO dataset	Cancer type	GENE (Probe ID)	*p*-value	Effect sign
GSE31210	Lung cancer	201251_AT	9.7e-05	Negative
GSE30929	Liposarcoma	201251_AT	0.000342	Negative
GSE11969	Lung cancer	3739	0.00194	Negative
GSE36471	Lung cancer	24498	0.00236	Negative
GSE13507	Bladder cancer	ILMN_1663122	0.00266	Positive
GSE16560	Prostate cancer	DAP3_2320	0.00488	Negative
GSE22762	Chronic lymphocytic leukemia	201251_AT	0.0126	Negative
GSE17536	Colon cancer	201251_AT	0.0195	Negative
GSE4271	High-grade glioma	201251_AT	0.0241	Negative
GSE22220	Breast cancer	2030537	0.0338	Negative
GSE24450	Breast cancer	ILMN_1672650	0.0428	Negative
GSE4573	Lung cancer	201251_AT	0.0448	Negative

### PKM2 exon-10 variants exhibit a reduced allosteric effect

To investigate the mechanistic basis of the exon-10 region involved in the allosteric effect of PKM2, we used tumor-related variants near the C–C interface: an oncogenic mutant R399E that promotes tumor growth in a mouse model^18^, a natural variant H391Y from Bloom syndrome patients with a risk of cancer^33,34^, and G415R found in tumor samples^35^. The purified G415R expressed in *Escherichia coli*, however, was quite unstable and exhibited essentially no detectable pyruvate kinase activity. The subsequent biochemical analyses were thus done for H391Y and R399E.

**Fig. 2:**
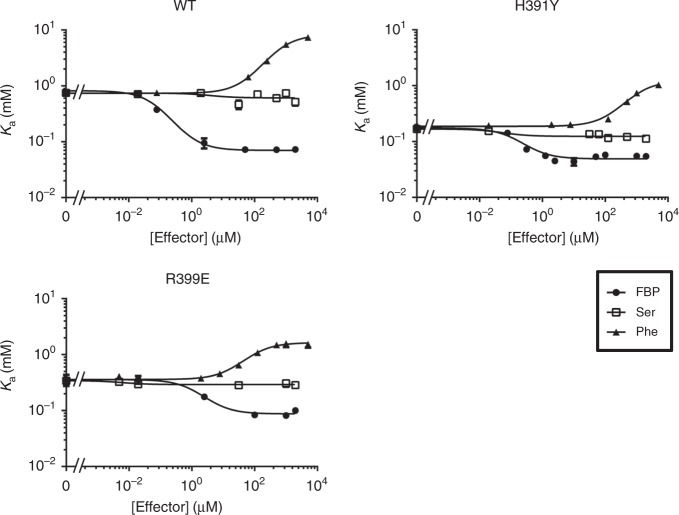
The coupling effect between the PEP binding affinity and an allosteric effector by the linked-function analysis. The profile of *K*_a_ values for PEP of PKM2 (wild-type, H391Y, or R399E) vs. an effector (FBP, Ser, or Phe) was measured. Data from three independent experiments are presented as mean ± SD. WT, wild-type

The apo-form H391Y and R399E had a slightly higher relative activity (163 and 137%) as compared with wild-type PKM2. We evaluated allosteric properties for H391Y and R399E by the linked-function analysis developed by the Reinhart’s laboratory^39^. The effect of an effector (FBP, Ser, or Phe) on the binding of phosphoenolpyruvate (PEP) for wild-type PKM2 was assessed by the plot of *K*_*a*_ for PEP as a function of a effector concentration. The derived values of coupling constant (*Q*_ax_) based on the linked-function equation are given in Table 2. As shown in Fig. 2, increasing concentrations of the activator FBP led to an evidently enhanced degree of the PEP binding affinity for wild-type PKM2, yielding a large value of *Q*_ax_ = 11.70. For Ser, there was a relatively horizontal profile and *Q*_ax_ = 1.22, suggesting that FBP is a much potent allosteric activator for the PEP binding affinity as compared with Ser. For the inhibitor Phe that binds at the same effector site of Ser, the magnitude of the inhibition was quite strong, generating a noticeably inverse trend and *Q*_ax_ < 1(0.09).

**Table 2:** Allosteric coupling constants of PKM2 variants

*Q*_ax_^a^	FBP	Ser	Phe
Wild-type	11.70 ± 2.62	1.22 ± 0.10	0.09 ± 0.01
H391Y	3.56 ± 0.22	1.35 ± 0.07	0.15 ± 0.01
R399E	4.10 ± 0.62	1.18 ± 0.07	0.22 ± 0.02

^a^Coupling constant (*Q*_ax_) is derived from the equation $K_{\mathrm{a}} = K_{{\mathrm{ia}}}^0\left[ {\left( {K_{{\mathrm{ix}}}^0 + \left[ {{\mathrm{PEP}}} \right]} \right)/\left( {K_{{\mathrm{ix}}}^0 + Q_{{\mathrm{ax}}}\left[ {{\mathrm{PEP}}} \right]} \right)} \right]$.

We next evaluated the allosteric properties per PKM2 variant. H391Y and R399E exhibited a reduced level of FBP activation profile (Fig. 2) and decreased values of *Q*_ax_ (3.56 and 4.10) (Table 2). For Ser, these variants displayed similarly horizontal patterns and comparable values of *Q*_ax_, indicating that Ser had a limited effect on the PEP binding affinity. By contrast, the inhibitor Phe inversely impacted the PEP binding affinity for H391Y and R399E (*Q*_ax_: 0.15, 0.22), albeit with a much lower effect as compared with wild-type PKM2. The apo-form H391Y and R399E displayed a slightly higher PEP binding affinity while considerably weaker coupling between FBP and PEP binding as compared with wild-type PKM2, indicating that binding affinity and allosteric effectiveness can be independent and uncorrelated to one another. A similar allosteric behavior is also reported for phosphofructokinase^40^. Together, our results suggest that FBP and Phe were prominent allosteric effectors and H391Y and R399E dramatically lost the allosteric properties.

### Structures of H391Y and R399E demonstrate altered conformer

**Fig. 3:**
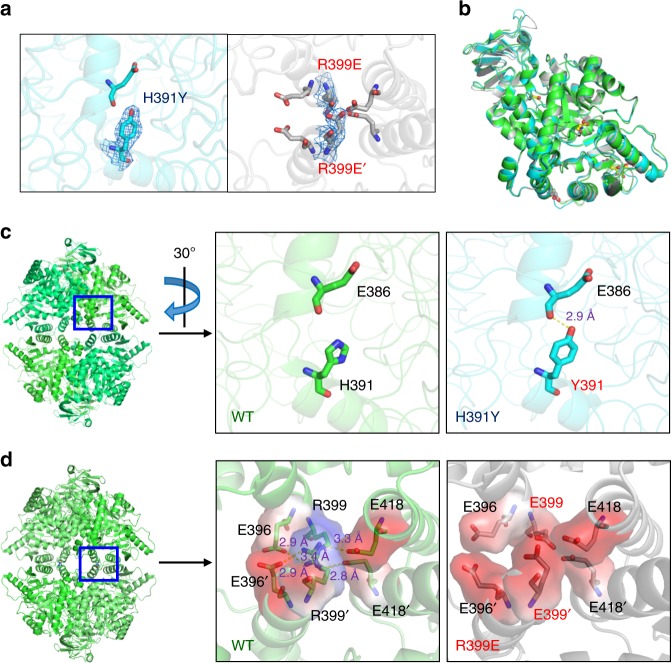
Structural analysis of PKM2 wild-type and variants. **a** The 2*F*_o_*-F*_c_ electron density map of H391Y and R399E. The map is contoured at the 1.1-σ level. **b** Superposition between wild-type (R-state, green), H391Y (cyan), and R399E (gray) monomers. Mutated residues are shown as heavy sticks. FBP, serine, and oxalate are drawn as ball-and-stick models. The carbon, oxygen, and nitrogen atoms are colored yellow, red, and blue, respectively. **c** Structural analysis of wild-type PKM2 and H391Y shows that H391Y possesses a strong hydrogen bond contact between Y391 and E386. The right panel is a zoomed region of 30 degree rotation clockwise of the left panel. **d** Comparison between wild-type PKM2 and R399E shows that R399E fails to form a salt-bridge network among R399, E396, and E418 at the C−C interface. The right panel is a zoomed region of the left panel. Residues (E386, H391, Y391, R399, E399, E396, and E418) are shown as sticks. The oxygen and nitrogen atoms are colored red and blue, respectively. wild-type (PDB: 3SRD; green); H391Y (PDB: 4YJ5, this study; cyan); and R399E (PDB: 5X0I, this study; gray)

The crystal structures of H391Y·Ser·FBP (*R* = 14.4%, *R*_free_ *=* 21.7%) and R399E·Ser·FBP (*R* = 18.0%, *R*_free_ = 21.8%) were determined through the molecular replacement method (Table 3). Figure 3a shows a clear electron density map of the substituted side chain for H391Y or R399E. The model consists of four subunits, and each monomer of H391Y and R399E displayed an overall homologous fold similar to that of the wild-type (Fig. 3b); each monomer (531 amino acids) comprised N region (1‒43), A domain (44‒116 and 219‒389), B domain (117‒218) and C domain (390‒531)^7^. The active site is situated at A domain in close proximity to B domain, and the FBP-binding (431‒437, 482, 489, 513‒522) and Ser-binding (43‒46, 70, 106, 464‒471) sites were situated in the C domain. Notably, an exclusive exon-10 region (378‒434) of PKM2 resides was observed near the intersubunit interface (A–A and C–C) (Supplementary Fig. 1). Table 3 summarizes the crystallographic statistics of PKM2 variants.

**Table 3:** Crystallographic statistics of PKM2 variants

PKM2	H391Y(Ser)	R399E(Ser)
PDB	4YJ5	5X0I
*Data collection*		
Space group	P2_1_	P2_1_2_1_2_1_
*Unit cell*		
*a* (Å)	73.22	116.60
*b* (Å)	140.89	137.88
*c* (Å)	108.72	149.71
Resolution (Å)	20.00–2.41	30.00–2.64
Unique reflections	83766	72242
Completeness (%)^a^	99.9 (99.9)	99.9 (100)
Average *I/σ (I)*^a^	17.0 (4.2)	16.6 (4.0)
Redundancy^a^	4.3 (4.4)	4.8 (5.0)
*R*_merge_(%)^a,b^	7.8 (39.1)	9.3 (49.7)
*Refinement*		
*R* value (%)^c^	14.4	18.0
*R*_free_ value (%)^d^	21.7	21.8
R.m.s.d. bond lengths (Å)^f^	0.015	0.016
R.m.s.d. bond angles (°)^f^	1.906	1.477
Ligands	FBP/Ser	FBP/Ser

^a^Values in parentheses refer to statistics in the highest-resolution shell

^b^*R*_merge_ = Σ|*I* − <I>|/Σ(*I*)

^c^*R* = Σ|*F*_obs_-*F*_calc_|∕Σ*F*_obs,_ where *F*_obs_ and *F*_calc_ are the observed and calculated structure-factor amplitudes, respectively

^d^*R*_free_ was computed using 5% of the data assigned randomly

The replacement of H391 with Y391 introduced a strong hydrogen bond contact between the hydroxyl group of Y391 and carboxyl group of E386 (3.0 Å), which strengthened the connection of the two helices (residues 371−388 and 391−401) near the C–C interface (Fig. 3c). The wild-type exhibited strong salt-bridge intersubunit interactions among R399, E396, and E418 at the C–C interface. However, substitution by E399 essentially forfeits these contacts because of an anionic carboxyl moiety, even in the presence of FBP, and thus a conformer that lost allosteric regulation (Fig. 3d). We could not obtain crystals of G415R given its instability. The structural analysis of G415 shows that G415 is situated at the middle of a long helix (residues 407−423) at the C–C interface. Replacement with arginine introduced a long, guanidine side chain at this position, which is presumed to perturb the helical conformation and tetrameric assembly. Collectively, these results suggest that the C–C interface variants exhibited altered structural elements that interfere with the conformational transformation into an active form and allostery.

### PKM2 exon-10 variants display increased interaction with KDM8

**Fig. 4:**
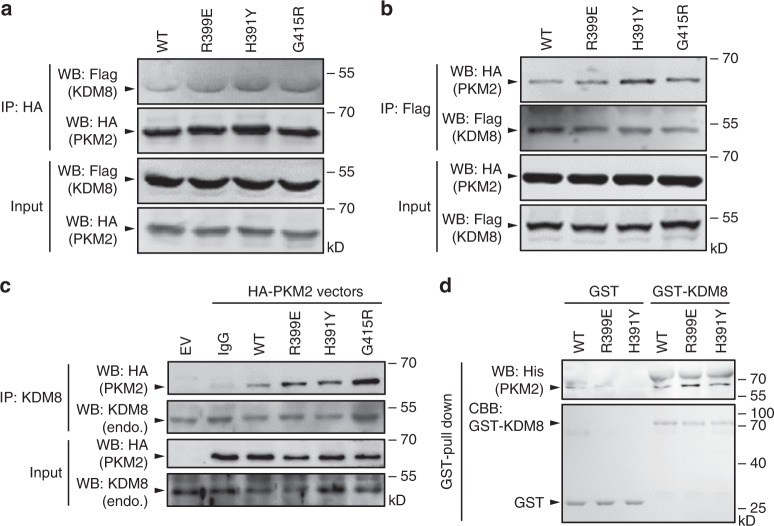
PKM2 exon-10 variants show increased interaction with KDM8. **a**, **b** Co-immunoprecipitation (IP) assay was conducted by co-transfecting MCF7 cells with Flag-KDM8 plus a HA-tagged PKM2 (HA-PKM2) protein as indicated, followed by IP with anti-HA (**a**) or anti-Flag (**b**) and Western blotting analysis. **c** IP assay was conducted by transfecting MCF7 cells with a HA-PKM2 as indicated, followed by IP with anti-KDM8 and the subsequent Western blotting analysis. **d** GST pull-down assays were performed with GST or GST-KDM8 and a His-tagged PKM2 (His-PKM2) protein as indicated. WB, Western blotting analysis; EV, empty vector; endo., endogenous; CBB, Coomassie brilliant blue; WT, wild-type

KDM8 makes direct partnership with PKM2 to promote its nuclear translocation and the subsequent transactivation event^21^. Since PKM2 interacts with KDM8 through its C-terminal domain^21^, we asked whether PKM2 exon-10 variants with altered C–C interface conformation affected the binding affinity with KDM8. We used co-IP experiment of lysates from MCF7 cells co-transfected with HA-tagged PKM2 (wild-type, R399E, H391Y, or G415R) and Flag-tagged KDM8. Each of PKM2s was immunoprecipitated using anti-HA antibodies, followed by Western blotting analysis. Figure 4a shows that all three exon-10 variants (R399E, H391Y, and G415R) exhibited higher affinity to KDM8 as compared with wild-type PKM2. The full-sized blot images are shown in Supplementary Fig. 2. A reciprocal immunoprecipitation (IP) assay using anti-Flag antibody to precipitate KDM8 also revealed stronger interaction with the PKM2 variants (Fig. 4b). In addition, the association of PKM2 mutants with endogenous KDM8 in MCF7 cells was studied. IP analysis using anti-KDM8 revealed that each mutant exhibited a higher degree of interaction with endogenous KDM8 than did wild-type PKM2 (Fig. 4c).

We next evaluated the KDM8-PKM2 interaction using cell-free pull-down assays with purified His-tagged PKM2 and GST-KDM8. Figure 4d shows that GST-KDM8 had stronger interaction with R399E and H391Y as compared with wild-type PKM2. Size-exclusion chromatographic analysis of purified recombinant proteins demonstrated that in the presence of KDM8, a higher proportion of PKM2 variants (R399E and H391Y) shifted to the position (12.5−13-mL fractions) with molecular masses corresponding to a dimeric (PKM2)_2_ or PKM2–KDM8 than did wild-type PKM2 (Supplementary Fig. 3). Thus, those exon-10 variants not only caused structural alterations near the C–C interface and lessened the allosteric regulation, but also interacted favorably with KDM8.

### PKM2 variants promote cell malignancy together with KDM8

**Fig. 5:**
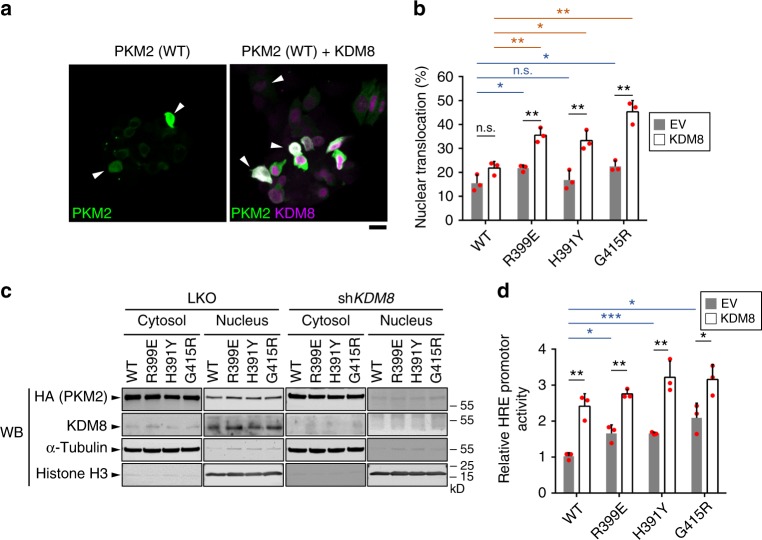
KDM8 promotes the nuclear translocation and transactivation activity of allostery-insensitive PKM2 variants. **a**, **b** MCF7 cells were transfected with HA-PKM2s (wild-type, R399E, H391Y, and G415R) or co-transfected with HA-PKM2 plus Flag-KDM8, followed by staining with anti-HA (HA-PKM2, green) and anti-Flag (Flag-KDM8, magenta). Representative images of the nuclear translocation of PKM2 in wild-type-PKM2-expressing cells in the absence or presence of KDM8 are shown in **a**. Arrowheads indicate the cells with detectable nuclear localization of PKM2. Bar 20 μm. The mean percentage of nuclear-localized PKM2 cells over PKM2-expressing cells from three preparations (*n* ≥ 50) for each PKM2 type (wild-type, R399E, H391Y, and G415R) was determined as shown in **b** (wild-type vs. R399E, *p* = 0.043; wild-type vs. H391Y, *p* = 0.040; wild-type + KDM8 vs. H391Y + KDM8, *p* = 0.015). **c** MCF7 cells were transfected with HA-PKM2s with or without knocking down endogenous KDM8 as indicated. Cells were then fractionated followed by Western blotting analysis. Histone H3 and α-tubulin were used as the marker for nucleus and cytosol, respectively. **d** Nuclear transactivation activity (wild-type vs. R399E, *p* = 0.013; wild-type vs. H391Y, *p* = 0.011; G415R vs. G415R + KDM8, *p* = 0.034). Statistical significance was evaluated using the two-tailed *t*-test. *p *<* 0.05; **p *<* 0.01; ***p *<* 0.001; n.s., *p* > 0.05; LKO, pLKO shRNA control. Bar plots are shown in mean ± SD. WT, wild-type

PKM2, primarily in the dimeric form, translocates into the nucleus and serves as a coactivator for transcriptional factors, including HIF1α, Stat3, β-catenin, and Oct-4^41^. We have previously demonstrated that KDM8 promotes the nuclear translocation of PKM2. Accordingly, we determined whether the exon-10 point-mutation variants altered the degree of KDM8-mediated translocation. We utilized confocal microscopic analysis to visualize the distribution of PKM2 and KDM8 in MCF7 cells transfected with PKM2 alone or PKM2 plus KDM8 (Supplementary Fig. 4). Cell numbers of at least 50 per assay were calculated to determine the level of nuclear translocation. Overexpression of mutant PKM2 alone led to an increased proportion of cells harboring nuclear PKM2 as compared with that of wild-type PKM2; R399E and G415R exhibited statistical significance (Fig. 5a, b). Remarkably, overexpression of KDM8 and each variant showed a significantly increased proportion of cells with nuclear PKM2 signal compared with that of PKM2 alone. There was also a slightly increased fraction of nuclear-PKM2 cells for cells co-transfected with KDM8 and wild-type PKM2 (Fig. 5b). We then surveyed the signal of nuclear PKM2 in those cells. Of note, the intensity of nuclear PKM2 in cells showing Flag-KDM8 signal (*n* = 7) was significantly higher with that in cells without Flag-KDM8 signal (*n* = 7) (Supplementary Fig. 5).

We next asked whether the depletion of KDM8 had an inverse effect by using control (LKO) and KDM8-depleted (sh*KDM8*) cells^21^. Analysis of nuclear and cytosolic fractions from lysates of LKO cells transfected with HA-PKM2 revealed that overexpression of each PKM2 mutant had a slightly higher level of nuclear PKM2 than did that of wild-type PKM2 (Fig. 5c). For sh*KDM8* cells, overexpression of wild-type and mutants had essentially no nuclear PKM2 signal detected, suggesting that KDM8 accelerated PKM2’s nuclear translocation.

To substantiate this notion, we measured the HIF-1 transactivation activity using a HIF1α-based reporter activity assay^21^. MCF7 cells were co-transfected with pHRE-FLuc, an internal control vector, and the empty vector or PKM2 (wild-type, R399E, H391Y, or G415R) in the absence or presence of KDM8 vectors. Figure 5d shows that cells co-expressing PKM2 and KDM8 had a significantly higher activity than did those expressing PKM2 alone. Furthermore, compared with wild-type PKM2 alone, a higher mean transactivation activity per variant was also observed.

**Fig. 6:**
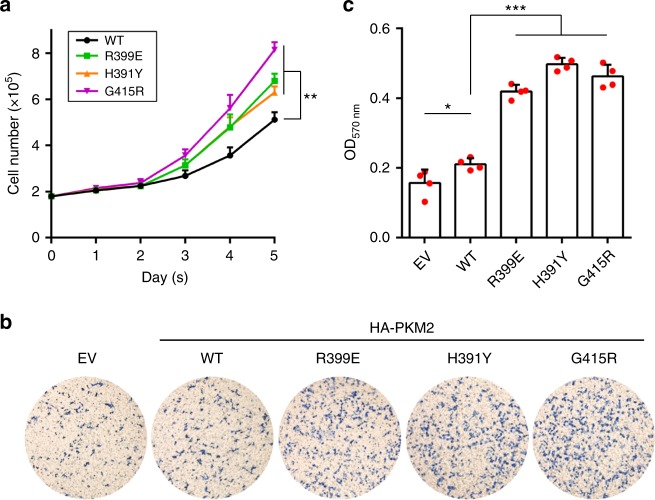
Overexpression of PKM2 exon-10 variants promotes cell proliferation and migration. **a**, **b** MCF7 cells were transfected with each HA-PKM2 mutant as indicated, followed by cell number counting over a five-day period (**a**) and migration assay (**b**). Representative images of migration are shown (**b**). **c** Quantitation of cell migration activity from **b** (EV vs. wild-type, *p* = 0.042). Statistical significance was evaluated using the *t*-test (two-tailed). *p *<* 0.05; **p *<* 0.01; ***p *<* 0.001. Plots are shown in mean ± SD. WT, wild-type

We then evaluated whether overexpression of PKM2 mutant can promote cancer progression. Figure 6a shows that overexpression of each mutant (R399E, H391Y, and G415R) in MCF7 cells had a significantly higher growth rate as compared with that of wild-type PKM2 over a five-day period. Additionally, compared with wild-type, each variant exhibited a significantly elevated level of migration (Fig. 6b, c). Together, these results suggest that the allostery-insensitive PKM2 variants confer increased regulation by KDM8 and promote aggressive cancer progression.

## Discussion

Taken together, we provided a key mechanistic basis of transformation of PKM2 into a signaling molecule through the structural changes near the C–C interface and hence allosteric resistance by characterization of PKM2-specific exon-10 variants (H391Y, R399E, and G415R). The coupling-function analysis revealed that wild-type PKM2 displayed a strong coupling effect between the PEP binding affinity and FBP. In contrast, there was a considerably weaker coupling effect for H391Y and R399E. A similarly inverse coupling pattern was seen for the other effector Phe. From the structural point of view, the large coupling effect between FBP and the PEP binding affinity for wild-type PKM2 is in good agreement with a large T-to-R conformational change induced by FBP so as to generate a much tighter substrate-binding environment for catalysis^7^.

PKM2 consists of the other Phe/Ser allosteric site distinct from the FBP site^10,42^. Of note, the PKM2-Phe complex structure is present as a T form (PDB: 4FXJ), while the Ser-liganded one (PDB: 4B2D) is an R form^10,42^. Superposition of PKM2-Phe and PKM2-Ser complexes reveals a larger pocket for the Phe-liganded complex. Since Phe possesses a bulky hydrophobic side chain, it is likely that binding of Phe to this site may induce an R-to-T conformational change, leading to an inverse coupling trend. On the other hand, Ser has a much smaller side chain and can fit quite well into this smaller pocket seen in the Ser-liganded structure. This might explain why Ser had a limited coupling effect.

The structures of H391Y and R399E clearly show an altered conformer at the C–C interface. H391Y is a considerably rigid conformer because of an additional strong hydorgen bond between two nearby helices, which is likely to greatly reduce the T-to-R conformational change and the allosteric regulation. R399E shows a diminished salt-bridge network near the C–C interface. As such, R399E exhibits an FBP-resistant allosteric activation as seen in the coupling-function analysis. Despite no G415R structure, the substitution of G415 with Arg situated in the middle of an intersubunit helix (405–423) may destabilize the dimer–dimer contacts, and this notion is supported by the observation of prompt aggregation after purification. Thus, those variants demonstrate altered conformation near the C–C interface and hence resistance to allosteric regulation.

PTM provides an alternative strategy to interefere with allosteric regulation of PKM2. For instance, an acetylated-K433 PKM2 or its acetylated mimic K433Q confers nonresponsiveness to FBP and exhibits a higher degree of nuclear transformation^29^. Phosphorylation of Y105 also resists to FBP-mediated activation^43^. Thus, those PKM2s with acquired resistant allostery, either from mutations or PTMs, are prone to transforming into a signaling molecule. Interestingly, the C-terminal segment consists of R445, R447, and R455 from PKM2 but not from PKM1 can be methylated by CARM1, which activates aerobic glycolysis but suppresses oxidative phosphorylation through regulating the expression and interaction with inositol 1,4,5-triphosphate receptors^44^. This finding reveals an additional layer of regulation for dimeric PKM2 that possibly has an exposed C-terminal region as opposed to PKM1. Thus, an endured or even a persistent dimeric/monomeric form of PKM2 not only curbs its pyruvate kinase activity but also is inclined to exposing the nuclear localization signal region^45^, a regulatory region^44^, or perhaps a peptide-interaction region near the C–C interface region given the lack of tetrameric assembly. This allows PKM2 interact with importin α5^15^ or a nuclear partner KDM8^21^ to facilitate nuclear translocation, or a subsequent methylation of the C-terminal segment to promote aerobic glycolysis^44^. Together, these results highlight the importance of endurance of a dimeric/monomeric PKM2 status through indolent allostery in cancer metabolism.

We also demonstrated an accelerated increase in nuclear translocation and transactivation activity in the presence of KDM8, an oncogenic histone demethylase^23^. We have previously reported that KDM8 is frequently overexpressed in several types of tumors; it heterodimerizes with PKM2, facilitates the nuclear translocation of wild-type, and therefore promotes HIF1α-mediated transactivation^21^. Here, the mutants displayed a higher level of nuclear translocation and HIF1α-mediated transactivation activity. It is noted that the two different approaches (confocal microscopic analysis and HRE promoter activity analysis) utilized here had a slight discrepancy in the case of overexpression of mutant PKM2 alone. Our speculation is that the endogenous amounts of KDM8 are not sufficiently enough to lead to a substantial degree of PKM2 nuclear translocation which can be evidently detected by confocal microscopic analysis. On the other hand, the HRE promoter activity assay that employs the reporter plasmid carrying 3 HRE repeats fused to firefly luciferase is much more sensitive, hence an increased level of HRE signal. We have previously mapped that the most critical KDM8 interaction region of PKM2 is situated at the C-terminal part (residues 366−476)^21^. Docking analysis also reveals that a monomeric PKM2 contacts with KDM8 through its C-terminal domain (Supplementary Fig. 6a). This interaction appears to disrupt the tetrameric form of PKM2, in favor of the “nuclear translocatable” dimer/monomer form (Supplementary Fig. 5) and facilitate hypoxia-inducible HIF1α-mediated transactivation. Our previous work also indicated that KDM8 catalytic mutant was able to enhance HIF1α-mediated transactivation via PKM2 translocation^21^, suggesting its activity is not required for this process. Interestingly, the analyses of TCGA and ICGC databases reveal numerous mutations clustered at the exon-10 region, in addition to G415R (V399M, R400H, E410K, A412V, V417L, A413V, V414M, and A427V) (Supplementary Fig. 6b)^25,26^, reflecting a potential selection advantage. It is thus likely that those exon-10 variants including H391Y, R399E, and G415R that bear the reduced-allostery trait confer the major proliferative advantage even in the absence of KDM8.

Based on the results of this study, we propose that the selection of allostery-resistant mutants at exon 10 provides an alternative pathway to enhance its non-metabolic function for a growth advantage to tumor cells. This reveals a new mutation-driven oncogenic feature of PKM2 mutations, in addition to non-enzymatically functional proteins resulting from truncated/frameshift mutations and missense mutations of PKM2, predicted to lower or reduce enzymatic activity^35^. These findings collectively strengthen the notion that PKM2 is a key isoform in cancer metabolism.

Previously, small-molecule PKM2 activators crosslinking the A–A interface to attain a constitutively active tetramer have been developed to suppress aerobic glycolysis to reduce the tumor growth^14^. Kung et al. reports that pharmacological activation of PKM2 using small-molecule activators induces serine auxotrophy for continued cell proliferation in A549^46^. This implies that PKM2 with tunable kinase activity regulates the metabolic state so as to manage the requirements of multiplying tumor cells and that cells persistently carrying high-activity PKM2 form might reroute its metabolism for sustained tumor growth. Instead, targeting the PKM2–KDM8 partnership in tumors carrying abundant expression of PKM2 and KDM8 represents a potential new intervention strategy. We have previously demonstrated that the knockdown of KDM8 compromised the growth of cancer cells^21–23^. Interestingly, a recent study reports that nuclear PKM2 can be substantially retained in the presence of the polymer of ADP-ribose (PAR) in EGFR-mutant cancers^47^. The depletion of PAR suppressed PKM2 nuclear localization and the cell growth of EGFR-mutant cancers^47^. By the use of the PC3 cell model, blocking the nuclear translocation of PKM2 using DASA-58 or metformin significantly impaired metastatic propagation in SCID mice^9^. These results suggest a new avenue for cancer interventions through the targeting of nuclear PKM2 or the PKM2–KDM8 partnership.

**Fig. 7:**
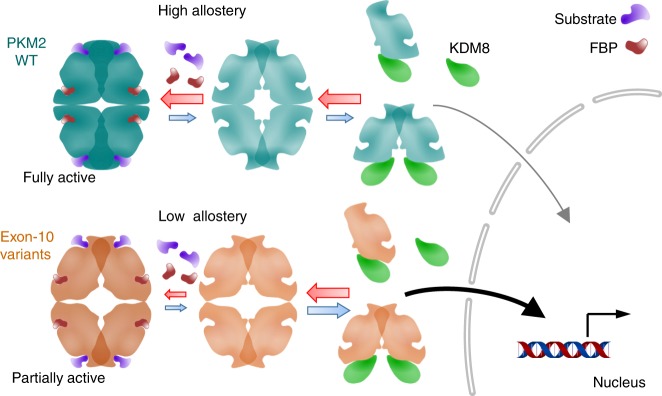
The proposed model that depicts the mechanistic basis of PKM2 exon-10 mutations in allosteric regulation and KDM8-mediated nuclear translocation. WT, wild-type

In sum, we demonstrate that tumor-related exon-10 mutations in PKM2 conferred altered conformation near the C–C interface region, a substantial decrease of allosteric regulation, and increased interaction with KDM8, facilitating nuclear translocation and transactivation (Fig. 7). This is particularly pronounced in the presence of KDM8, an oncogenic partner highly overexpressed in breast tumors. These results add a mutation-driven strategy to transform PKM2 into a signaling molecule, which can rewire cell metabolism to meet the special metabolic demands of proliferating tumor cells in appropriate context. Targeting the PKM2-KDM8 complex may provide a potential therapeutic intervention of PKM2-associated tumors.

## Methods

### Plasmid construction

PKM2 full-length cDNA was amplified by PCR using DNA polymerase (SMOBIO Technology, Inc.) and dNTP (Genedirex, Las Vegas City, NV, USA) and ligated to pET28a (Novagen) vector for *E. coli* expression. All PKM2 mutants were generated by site-directed mutagenesis method. For eukaryotic cell expression, PKM2 wild type and mutant cDNAs were inserted into pcDNA3.1 vector. The primer sequences used for PCR amplification are shown in Supplementary Table 1. All sequences were verified by DNA sequencing (Supplementary Data 2).

### Expression and purification of PKM2

Protein expression of *E. coli* BL21 (DE3) carrying desired PKM2 wild type/mutant plasmids were induced by 1.0 mM IPTG (isopropyl-β-d-thiogalactopyranoside) under 16 °C incubation. Cells were harvested and homogenized by sonication. After centrifugation (10,000 × *g* at 4 °C for 20 min), the recombinant protein was purified from crude extract by using cobalt-chelated TALON Metal Affinity Resin (Clontech) under the manufacturer’s instruction. Eluted protein was concentrated and dialyzed into Tris buffer (40 mM Tris, 100 mM KCl, pH 7.5) using a Amicon Ultra-15 30,000 M.W. tube (Millipore). Protein purity was checked by SDS-PAGE followed by Coomassie brilliant blue staining.

### Measurement of PKM2 activity and allostery

The in vitro PKM2 pyruvate kinase activity was determined by measuring OD_340_ of lactate dehydrogenase-coupled reaction. A steady-state kinetic reaction was carried out in 50 mM Tris (pH 7.5), 100 mM KCl, 5 mM MgCl_2_, 1 mM ADP, 0.4 mM NADH, 2 U LDH, 25 ng PKM2 and the [PEP] in the range of 0.06–8 mM in total volume of 200 μL at 37 °C. Kinetic parameters were obtained using nonlinear regression fitting to the Michaelis–Menten equation; the errors in the parameters were less than 5%. Coupling constant (*Q*_ax_) which describes the effect of an effector (FBP, Ser, and Phe) on the binding of PEP is derived from the linked-function analysis $K_{\mathrm{a}} = K_{{\mathrm{ia}}}^0\left[ {\left. {\left( {K_{{\mathrm{ix}}}^0 + [{\mathrm{PEP}}]} \right)/K_{{\mathrm{ix}}}^0 + Q_{{\mathrm{ax}}}\left[ {{\mathrm{PEP}}} \right]} \right)} \right]$^39^, where *K*_a_ is dissociation constant defined as the concentration of substrate that yields a rate equal to one-half of the *V*_max_, $K_{{\mathrm{ia}}}^0$ is the dissociation constant for PEP in the absence of an allosteric effector, and $K_{{\mathrm{ix}}}^0$ is the dissociation constant for an allosteric effector in the absence of PEP. The kinetics data were collected by using CLARIOstar V5.01 R4 software. Coupling constants were analyzed by using GraphPad Prism 6.01.

### Size-exclusion chromatography

Size-exclusion chromatography was carried out using ÄKTA FPLC system. PKM2 protein was aliquot to 100 μL at the concentration of 2.5 mg/mL in PBS in the absence or presence of appropriate amounts per allosteric effector and run onto the Bio-Rad ENrich™ SEC 650 column equilibrated with PBS at the flow rate of 1 mL/min. The elution process was monitored by OD_280_, and eluted protein was collected with 0.5 mL fraction size.

### Protein crystallization

Initial crystallization screening was carried out by using the Oryx8 robot (Douglas Instruments, UK). Subsequent crystal optimization was performed by mixing equal volumes of a protein sample (8.0 mg/mL PKM2) and the reservoir solution (0.1 M Tris, pH 7.5, 0.2 M NaCl, 14% w/v polyethylene glycol 3350) using hanging drop vapor diffusion method at 4 °C.

### X-ray data collection and structure refinement

X-ray diffraction data of H391Y·Ser·FBP and R399E·Ser·FBP structures were collected at National Synchrotron Radiation Research Center (NSRRC, Hsinchu, Taiwan) using ADSC Quantum-315r CCD area detector. H391Y·Ser·FBP and R399E·Ser·FBP crystals were freshly mounted at BL13B1 and BL13C1 beamlines, respectively. All dataset integration and scaling were calculated using HKL-2000. Data collection statistics are shown in Table 3.

The PKM2 structure models were constructed by using Molrep program in CCP4 interface (version 2.2.1). The starting model for molecular replacement was wild type PKM2 structure from Protein Data Bank (PDB code: 4B2D). Structure refinement was done by using the REFMAC5 program in CCP4 interface. Manual refinement of the structures was done by using COOT (version 0.7.2) program.

### Structural modeling of the PKM2-KDM8 complex

The PKM2–KDM8 complex model was generated by the ZDOCK module of the Discovery Studio 2017 client (Accelrys Inc., USA). The input PKM2 and KDM8 coordinates were extracted from the structures of PKM2 (PDB code: 3SRD)^10^ and KDM8 (PDB code: 4GJY)^48^, respectively. The identified interaction regions in PKM2 and KDM8^21^ were selected as the binding sites. All generated poses were ranked according to the spatial proximity and the energy by the ZDock and ZRank scoring functions. The top-ranking poses were refined and optimized by refined docked proteins (RDOCK).

### GST pull down assay

His-tagged PKM2 (2 μM) and GST-tagged KDM8 (1 μM) or GST alone (1 μM) was incubated with pre-equilibrated GST beads (Glutathione Sepharose, GE) in Tris buffer [40 mM Tris, 100 mM NaCl, 0.5% Nonidet P-40, pH 8.0, and 1× protease inhibitor cocktail (Thermo)] for 2 h under 4 °C with agitation. The GST beads were centrifuged down and washed with the Tris buffer for three times. The samples were subject to 10% SDS-PAGE separation, followed by Coomassie brilliant blue staining and Western blotting analysis. Anti-His antibody (1:2000, CusAb) was used for the immunoblotting.

### Co-immunoprecipitation assay

MCF7 cells were purchased from the Bioresource Collection and Research Center of Food Industry Research and Development Institute, Taiwan. Cells harvested from culture dishes were lysed in IP lysis buffer [50 mM Tris-HCl (pH 7.4), 150 mM NaCl, 0.5% Nonidet P-40, 5% glycerol, 1 mM EDTA, and 1× protease inhibitor cocktail (Thermo)], followed by centrifugation (16,000 × *g*, 4 °C, 10 min) to remove insoluble debris. The lysates were incubated with 1 μg anti-HA or anti-Flag antibodies (Cell Signaling) and 10 μL of protein A/G magnetic beads (Invitrogen) at 4 °C overnight with agitation. Homemade rabbit anti-KDM8 antibody was used to precipitate endogenous KDM8. The beads were washed with IP lysis buffer for three times and subject to separation by 10% SDS-PAGE, followed by Western blotting analysis using the antibodies as indicated.

### Confocal microscopic analysis

MCF7 cells were fixed with 3.7% paraformaldehyde for 20 min and penetrated by treatment with blocking buffer (2.5% fetal bovine serum in PBS) containing 0.1% of Triton X-100 for 30 min. HA-PKM2 and Flag-KDM8 were marked with rabbit anti-HA-tag (1:500, SignalChem) and mouse anti-Flag-tag (1:500, Sigma-Aldrich), respectively. Cell nuclei were stained with Hoechst 33342. Alexa-488–conjugated anti-rabbit (1:500) and Cy5-conjugated anti-mouse (1:500) IgGs were used as secondary antibodies (Jackson ImmunoResearch Laboratories). Cells were examined in a Zeiss LSM 780 laser-scanning microscope with a Plan-Apochromat 20×/0.8 M27 objective. Images were analyzed using the LSM 780 META ZEN 2011 software package (Carl Zeiss). Mean fluorescent intensity was analyzed by using imageJ software. Statistical analysis was done by using Microsoft Excel 2016.

### Lentivirus production

HEK293T cells were seeded onto 10-cm culture dishes. The cells were transfected one day later by using Lipofectamine^TM^ 2000 (invitrogen) with lentiviral transducing vector encoding pLKO or sh*KDM8* and packaging vectors. After 72 h, virus particles were harvested from the medium and filtered through a 0.45-mm syringe-driven filter (Millipore).

### Subcellular fractionation

MCF7 cells were scrapped from tissue culture plates and lysed in hypotonic buffer (20 mM Tris, pH 7.4, 10 mM NaCl, 3 mM MgCl_2_) for 15 min on ice. Mild detergent (Nonidet P-40) was added to a final concentration of 0.5%. The nuclear fraction was precipitated by centrifugation at 800 × *g* for 10 min at 4 °C. The supernatant was collected as the cytosolic fraction, and the pellet was further lysed in complete lysis buffer (40 mM Tris, pH 7.4, 100 mM NaCl, 0.5% Nonidet P-40) to serve as the nuclear fraction.

### Luciferase activity assay

Cells were transfected with pHRE-Firefly luciferase reporter, internal control reporter pTK (Thymidine kinase)-Renilla luciferase (Promega), pcDNA3.1-HA control, per PKM2 vector (pcDNA3.1-HA-PKM2, pcDNA3.1-HA-R399E, pcDNA3.1-HA-H391Y, or pcDNA3.1-HA-G415R) or plus pcDNA3.1-Flag-KDM8 in 48-well plates. Firefly and Renilla luciferase activities were measured by using the Dual- Luciferase Assay System (Promega) following the manufacturer’s instruction.

### Cell proliferation assay

MCF7 cells were seeded in six-well plates (3 × 105 cells per well) and transfected with each PKM2 plasmid indicated. The cell number was counted each day in a five-day interval. Data represent the mean ± SD of three independent replicates.

### In vitro migration assay

For the migration/chemotaxis assays, we used cell migration kit (CytoSelect, CBA-100). In brief, cells were transfected with the indicated vectors and recovered for 24 h. Cells (2 × 105) were suspended in serum-free medium and seeded on top of the membrane. Serum-containing medium was placed at the bottom and cells that had migrated to the lower surface of the polycarbonate membrane were stained and counted after 16 h. A colorimetric approach by measuring OD 560 nm was used for quantification.

### Antibodies

Anti-HA antibody used in the immunoprecipitation experiment was purchased from Cell Signaling (catalog number: C29F4; lot number: 8). Anti-HA antibody used in the confocal microscopy analysis was purchased from SignalChem (catalog number: H98-63R; lot number: Q356-1). Anti-flag antibody was purchased from Sigma (catalog number: F1804; lot number: SLBS3530V). Anti-His antibody was purchased from CusAb (catalog number: CSB-MA000011M0m; lot number: D0612; clone number: 3G5). Alexa 488-conjucated anti-rabbit (catalog number: 111-545-003; lot number: 116143) and Cy5-conjugated anti-mouse (catalog number: 115-585-062; lot number: 122994) IgGs were purchased from Jackson ImmunoResearch Laboratories, Inc. The homemade rabbit anti-KDM8 antibody was raised against a full length of recombinant KDM8 protein.

### Reporting summary

Further information on experimental design is available in the Nature Research Reporting Summary linked to this article.

## Supplementary information

Supplementary Information

Description of Additional Supplementary Files

Supplementary Data 1

Supplementary Data 2

Reporting Summary

## Data Availability

The datasets analyzed during the current study are available in The Human Protein Atlas project (http://www.proteinatlas.org/), OncomineTM (http://www.oncomine.org/), and The Cancer Genome Atlas (TCGA, http://www.cbioportal.org/) databases. Coordinates of the PKM2-H391Y and PKM2-R399E structures have been deposited in the RCSB PDB with ID 4YJ5 and 5X0I, respectively. The plasmids reported in this study have been submitted to Addgene (deposit number 76407).
