# Supplementary material for: Mutations in the PKM2 exon-10 region are associated with reduced allostery and increased nuclear translocation
Source: Commun Biol. 2019 Mar 15;2:105. doi: 10.1038/s42003-019-0343-4 (PMC6420622; doi:10.1038/s42003-019-0343-4)
Supplement: Supplementary file 1 — Supplementary Information [file 42003_2019_343_MOESM1_ESM.pdf]

Supplementary Figures

Supplementary Figure 1

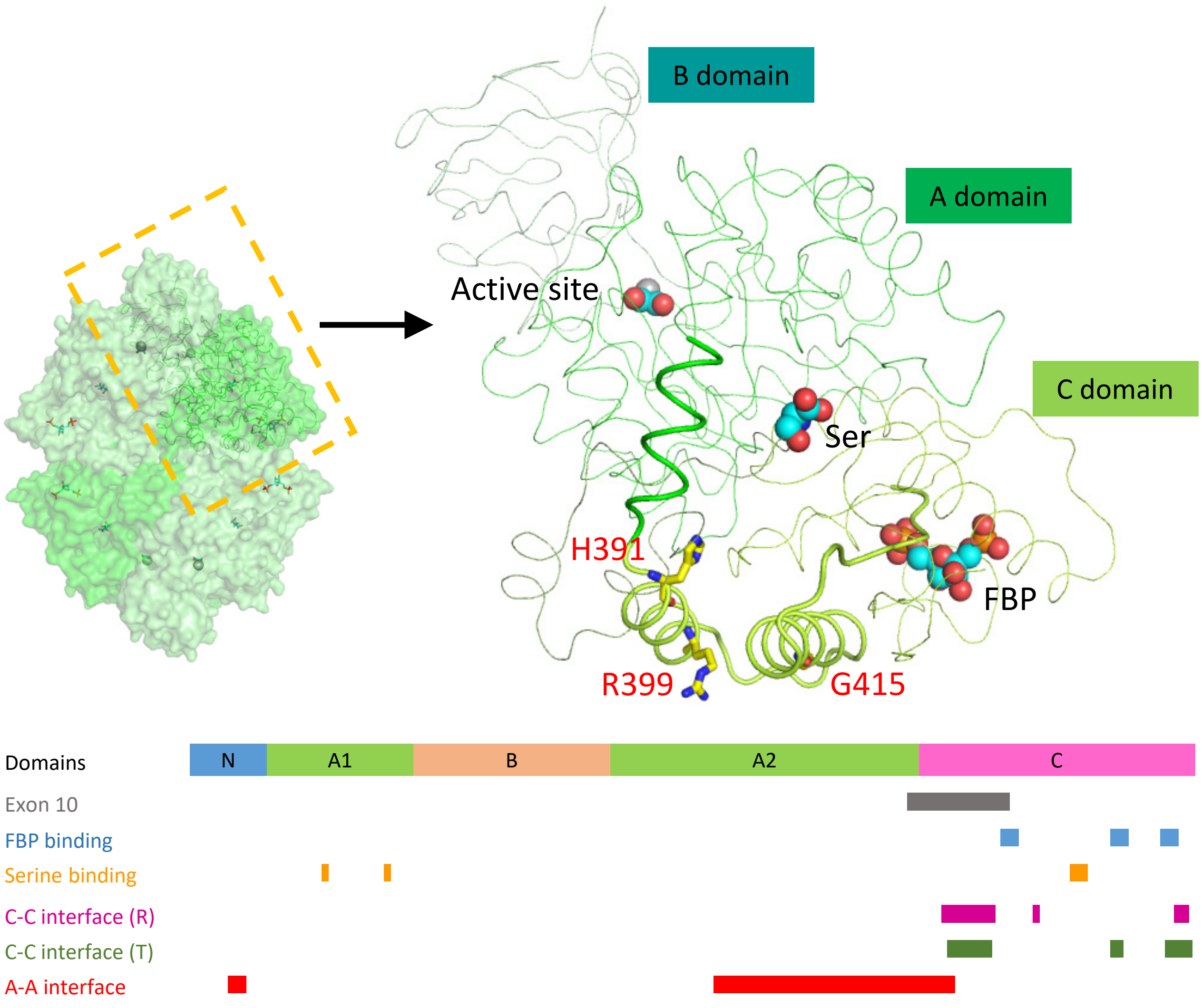

Supplementary Figure 1. Structure of PKM2. PKM2 is a tetramer in the crystal (left panel). Each monomer consists of an N region (1–43) and A (A1, 44–116; A2, 219–389), B (117–218), and C (390–531) domains. The A domain consists of the active site. The C domain contains the FBP-binding (431–437, 482, 489, 513–522) and Ser-binding (43–46, 70, 106, 464–471) sites. Allosteric regulation leads to a dramatic structural alteration in the C–C interface (R state: 395–424, 444, 523–527; T state: 399–423, 482–487, 515–529). The region of exon-10 residues is shown by a thick ribbon, in which H391Y, R399E, and G415R are indicated by stick models.

Supplementary Figure 2

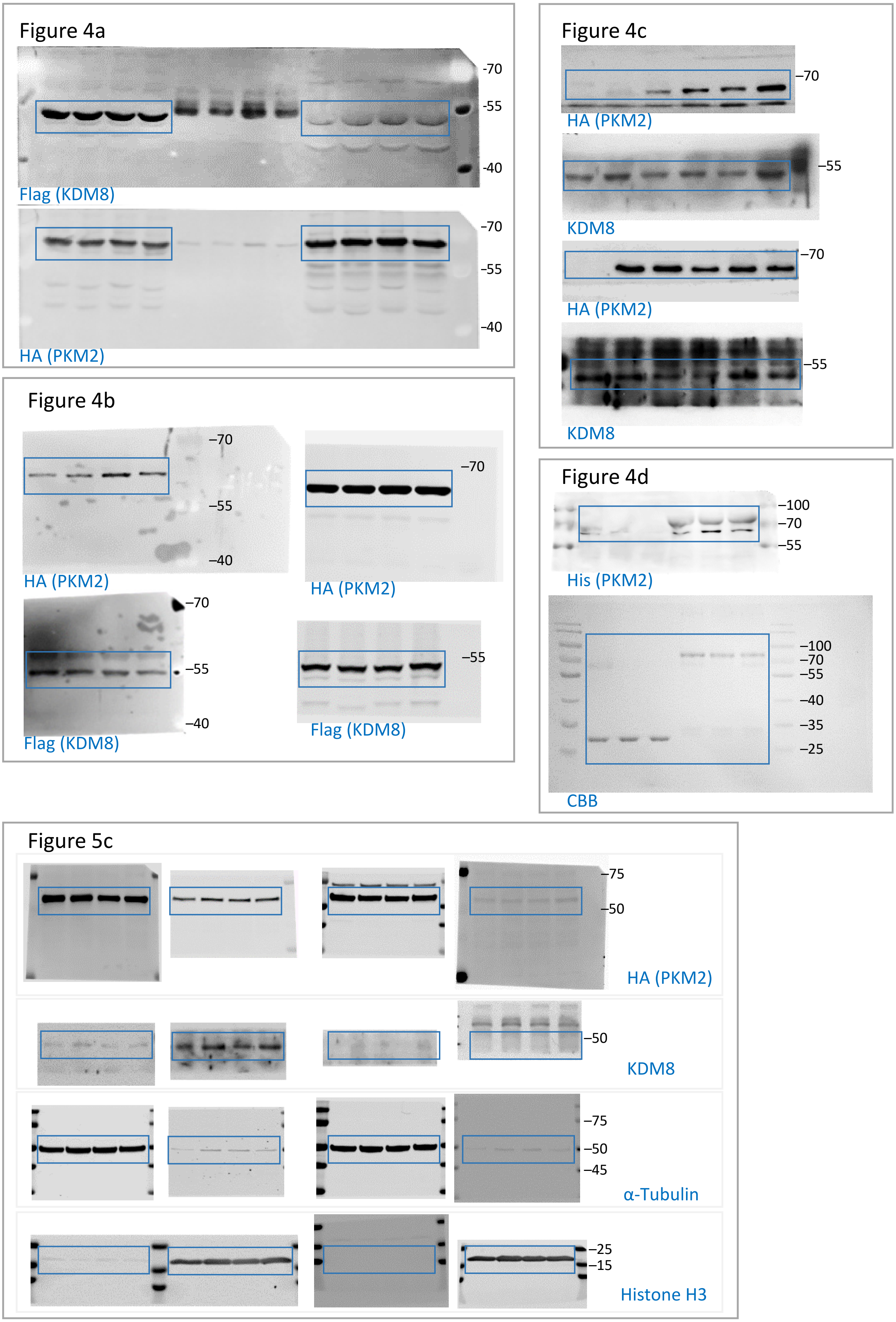

Supplementary Figure 2. The uncropped blot images. The molecular weight (kD) of the markers are shown as indicated.

Supplementary Figure 3

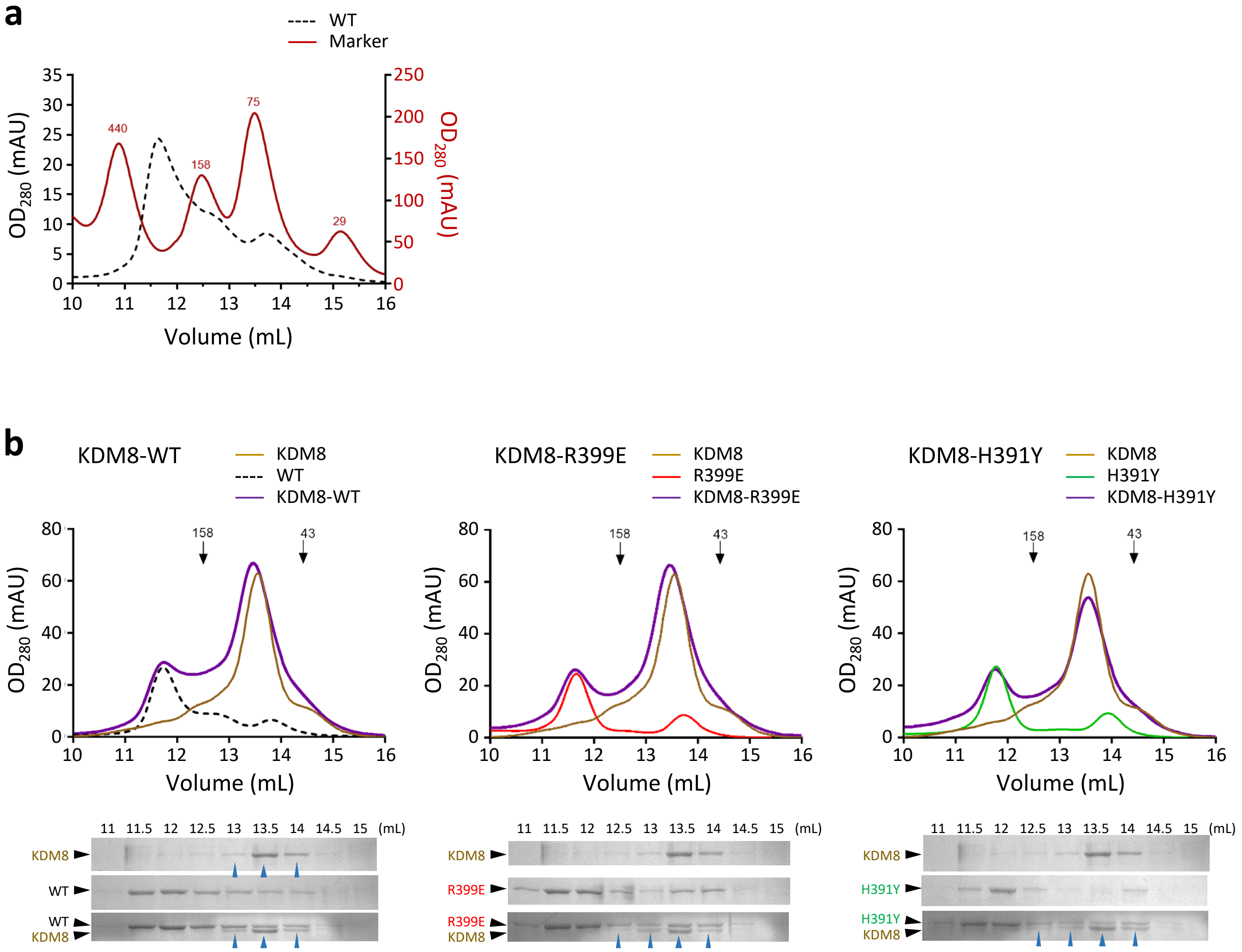

Supplementary Figure 3. Size exclusion chromatographic profiling of PKM2s in the absence or presence of KDM8. a The elution profile of PKM2 WT protein. The molecular weight (kD) of standard markers are shown on top per peak. **b** The 100-μL protein solution consisted of per PKM2 (WT, H391Y, or R399E) alone (2.5 mg/mL; dashed line), KDM8 alone (2.5 mg/mL; brown line) or both (2.5 mg/mL; purple line) was loaded onto Enrich™ SEC 650 column. The oligomeric status of eluted fractions (PBS solution; flow rate, 1 mL/min) was analyzed by SDS-PAGE gels (lower panel), followed by Coomassie brilliant blue staining. SDS-PAGE, sodium dodecyl sulfate-polyacrylamide gel electrophoresis; PBS, phosphate-buffered saline.

Supplementary Figure 4

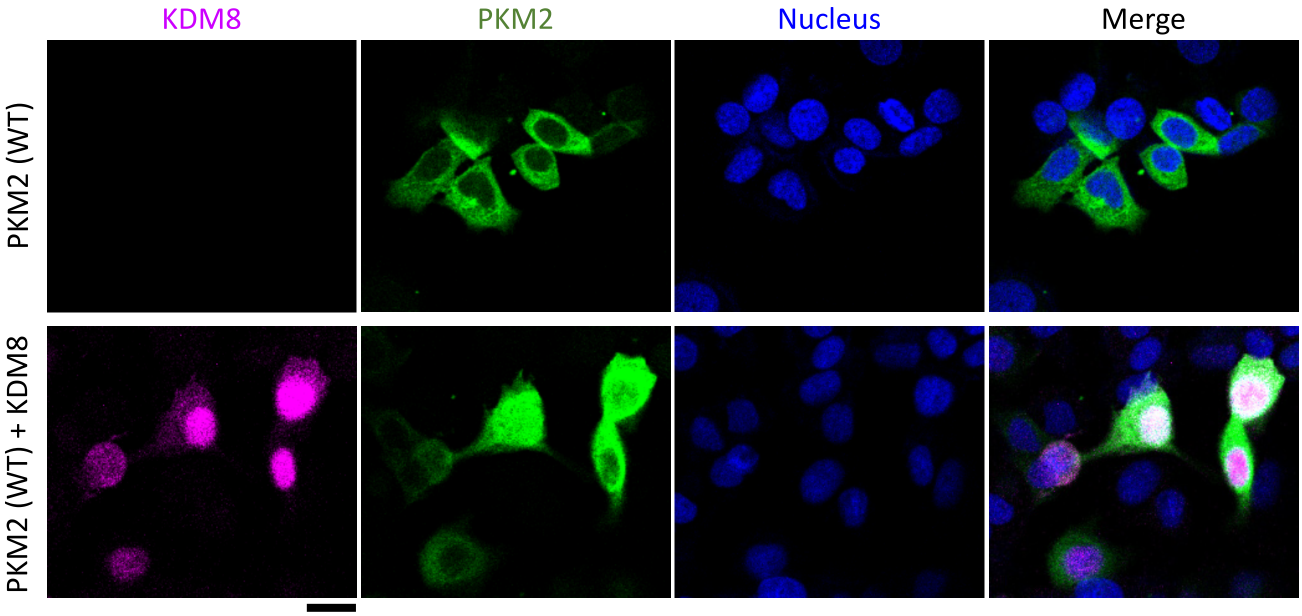

Supplementary Figure 4. Confocal microscopic analysis of nuclear-localized PKM2 regulated by KDM8 in MCF7 cells. MCF7 cells were transfected with HA-PKM2 or co-transfected with HA-PKM2 and Flag-KDM8, followed by staining with anti-HA (HA-PKM2, green) and anti-Flag (Flag-KDM8, magenta). The nucleus is marked with Hoechst (blue). Merged images (Merge) are shown. Bar, 20 μm.

Supplementary Figure 5

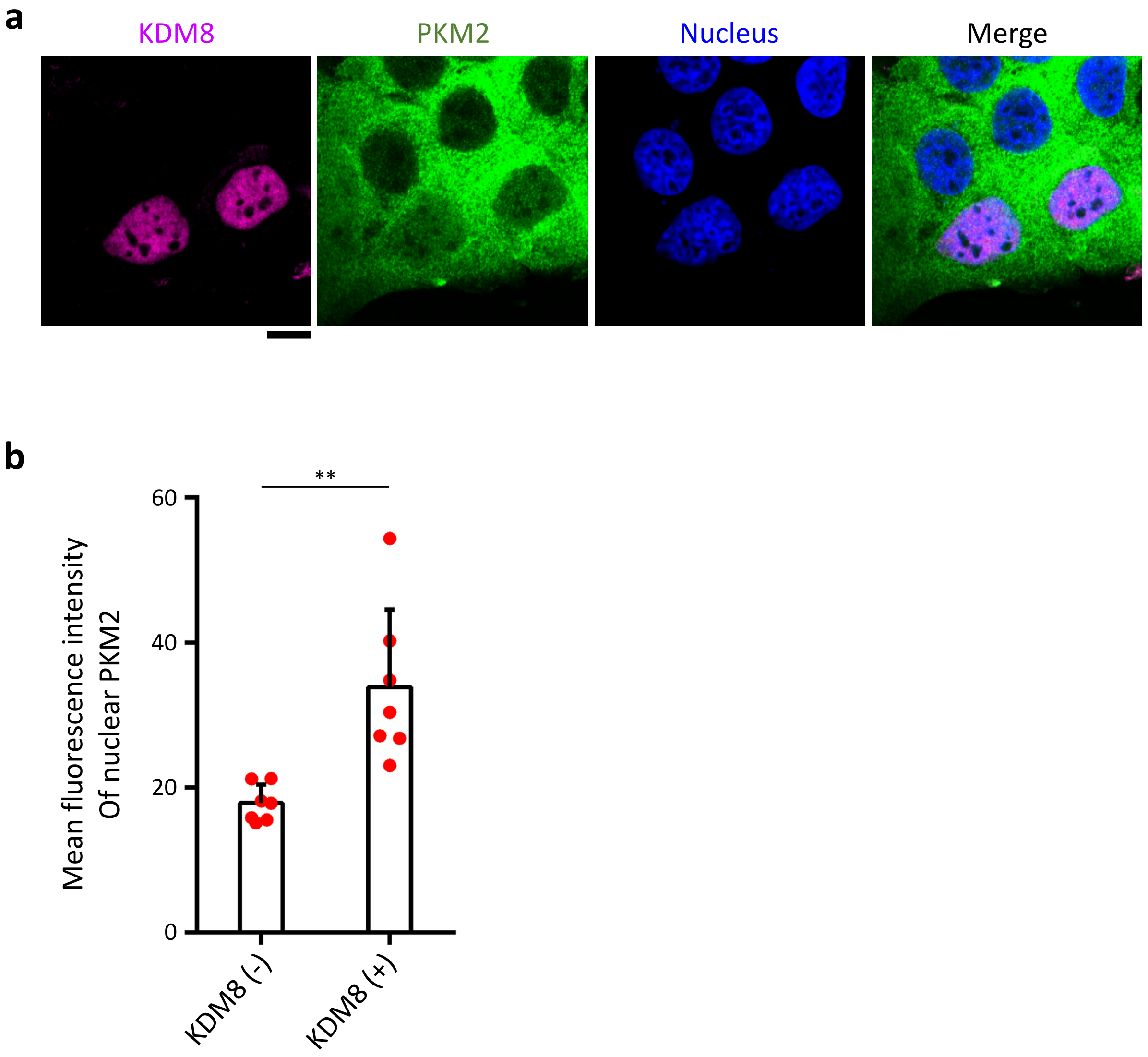

Supplementary Figure 5. Confocal microscopic analysis of PKM2 nuclear translocation regulated by KDM8 in MCF7 cells. a MCF7 cells were co-transfected with HA-PKM2 (WT) and Flag-KDM8, followed by staining with anti-PKM2 (green) and anti-Flag (Flag-KDM8, magenta). The nucleus is marked with blue color. Merged images (Merge) are shown. Bar, 10 µm. **b** Comparison of the nuclear fluorescence intensity of PKM2 between the KDM8 positive (+) and negative (-) cells. (n = 7 from three independent images, p = 0.002, two-tailed t-test). Bar plot is shown in mean ± SD.

Supplementary Figure 6

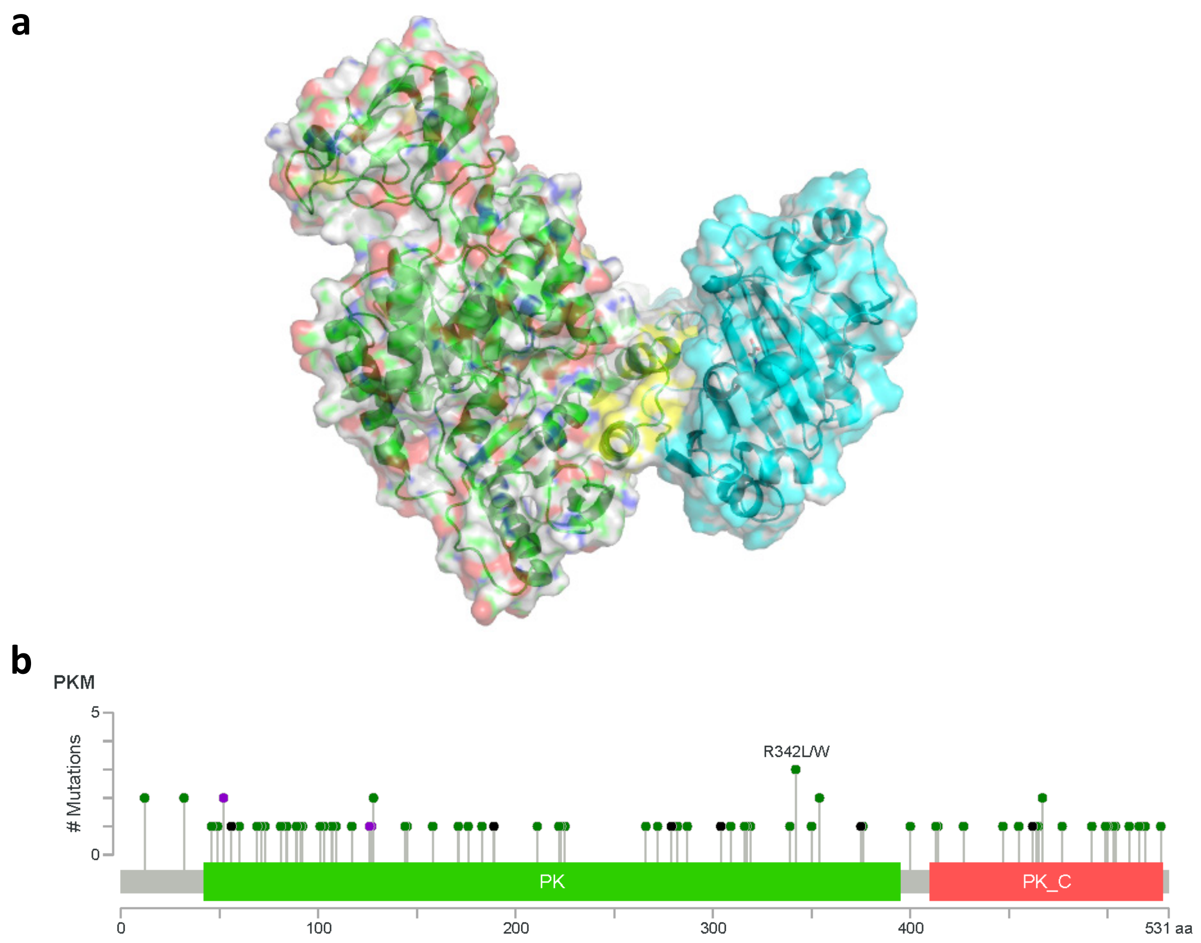

Supplementary Figure 6. The PKM2 docked model and somatic mutations from TCGA database. a A PKM2-KDM8 docked model based on the defined interaction region of PKM2 at the C-terminal region (residues 366–476). **b** Somatic mutations of PKM in TCGA database. Mutations of PKM2 in C domain are clustered near FBP-binding, Ser-binding regions, and the C–C interface. Green, missense mutation; black, truncating mutation; purple, other mutation.

Supplementary Tables

Supplementary Table 1 Primer sequences for plasmid construction.

Oligo	Oligo sequences (5’ to 3’)
PKM2 Forward	AAACATATGTCGAAGCCCCATAGTGAAGCC
PKM2 Reverse	AAAGGATCCTCACGGCACAGGAACAAC
PKM2 R399E Forward	GAGGAACTCGAGCGCCTGGCG
PKM2 R399E Reverse	CGCCAGGCGCTCGAGTTCCTC
PKM2 H391Y Forward	GCAGAGGCTGCCATCTACTATTTGCAA
PKM2 H391Y Reverse	TTGCAAATAGTAGATGGCAGCCTCTGC
PKM2 G415R Forward	CCGCCGTGCGTGCCGTGGAG
PKM2 G415R Reverse	CTCCACGGCACGCACGGCGG
KDM8 Forward	GACCTAAGCTTATGGCTGGAGACACCCAC
KDM8 Reverse	CTTAACTCGAGCGACCACCAGAAGCTGAC
