# Supplementary material for: Mutations in the PKM2 exon-10 region are associated with reduced allostery and increased nuclear translocation
Source: Commun Biol. 2019 Mar 15;2:105. doi: 10.1038/s42003-019-0343-4 (PMC6420622; doi:10.1038/s42003-019-0343-4)
Supplement: Supplementary file 2 — Description of Additional Supplementary Files [file 42003_2019_343_MOESM2_ESM.docx]

**Description of Additional Supplementary Items**

**File Name**: Supplementary Data 1

**Description**: The source data of graph/chart figures.

**File Name**: Supplementary Data 2

**Description**: The sequencing result of the plasmids.
