# Supplementary material for: Mutations in the PKM2 exon-10 region are associated with reduced allostery and increased nuclear translocation
Source: Commun Biol. 2019 Mar 15;2:105. doi: 10.1038/s42003-019-0343-4 (PMC6420622; doi:10.1038/s42003-019-0343-4)
Supplement: Supplementary file 5 — Reporting Summary [file 42003_2019_343_MOESM5_ESM.pdf]

nature research

Corresponding author(s): Dr. Wen-Ching Wang

Reporting Summary

Nature Research wishes to improve the reproducibility of the work that we publish. This form provides structure for consistency and transparency in reporting. For further information on Nature Research policies, see Authors & Referees and the Editorial Policy Checklist.

Statistical parameters

When statistical analyses are reported, confirm that the following items are present in the relevant location (e.g. figure legend, table legend, main text, or Methods section).

n/a	Confirmed	
☐	☒	The exact sample size (*n*) for each experimental group/condition, given as a discrete number and unit of measurement
☐	☒	An indication of whether measurements were taken from distinct samples or whether the same sample was measured repeatedly
☐	☒	The statistical test(s) used AND whether they are one- or two-sided<br>*Only common tests should be described solely by name; describe more complex techniques in the Methods section.*
☒	☐	A description of all covariates tested
☒	☐	A description of any assumptions or corrections, such as tests of normality and adjustment for multiple comparisons
☐	☒	A full description of the statistics including central tendency (e.g. means) or other basic estimates (e.g. regression coefficient) AND variation (e.g. standard deviation) or associated estimates of uncertainty (e.g. confidence intervals)
☐	☒	For null hypothesis testing, the test statistic (e.g. *F*, *t*, *r*) with confidence intervals, effect sizes, degrees of freedom and *P* value noted<br>*Give P values as exact values whenever suitable.*
☒	☐	For Bayesian analysis, information on the choice of priors and Markov chain Monte Carlo settings
☒	☐	For hierarchical and complex designs, identification of the appropriate level for tests and full reporting of outcomes
☒	☐	Estimates of effect sizes (e.g. Cohen's *d*, Pearson's *r*), indicating how they were calculated
☐	☒	Clearly defined error bars<br>*State explicitly what error bars represent (e.g. SD, SE, CI)*

Our web collection on statistics for biologists may be useful.

Software and code

Policy information about availability of computer code

Data collection	Pyruvate kinase kinetics data were collected by using CLARIOstar V5.01 R4 software. Crystallographic data were collected at BL13B1 and BL13C1 facilities at National Synchrotron Radiation Research Center (NSRRC), Taiwan.
Data analysis	METABRIC data analysis was performed by using SPSS 12.0 software. Crystallographic data were processed by HKL2000 software. Enzyme kinetics, nuclear translocation, and transactivation figures were analyzed by using GraphPad Prism 6.01 software. Mean fluorescent intensity of confocal images was analyzed by using imageJ software.

For manuscripts utilizing custom algorithms or software that are central to the research but not yet described in published literature, software must be made available to editors/reviewers upon request. We strongly encourage code deposition in a community repository (e.g. GitHub). See the Nature Research guidelines for submitting code & software for further information.

Data

Policy information about availability of data

All manuscripts must include a data availability statement. This statement should provide the following information, where applicable:

- Accession codes, unique identifiers, or web links for publicly available datasets
- A list of figures that have associated raw data
- A description of any restrictions on data availability

The datasets analyzed in this investigation are retrieved from those deposited in The Human Protein Atlas (http://www.proteinatlas.org/), OncomineTM (http:// www. oncomine.org/), and The Cancer Genome Atlas (TCGA, http://www.cbioportal.org/). Coordinates of the PKM2-H391Y and PKM2-R399E structures have been deposited in the RCSB PDB with ID 4YJ5 and 5X0I, respectively.

Field-specific reporting

Please select the best fit for your research. If you are not sure, read the appropriate sections before making your selection.

☒ Life sciences ☐ Behavioural & social sciences ☐ Ecological, evolutionary & environmental sciences

For a reference copy of the document with all sections, see nature.com/authors/policies/ReportingSummary-flat.pdf

Life sciences study design

All studies must disclose on these points even when the disclosure is negative.

Sample size	For the nuclear translocation analysis of PKM2 variants, MCF-7 cells were seeded in 6-well plates in three independent replicates per PKM2 variant. Cells were fixed and stained for confocal microscopy analysis. Cell numbers of at least 50 (50-200) per assay were calculated to determine the level of nuclear translocation.
Data exclusions	No data were excluded from the analysis in this study.
Replication	All measurements were performed in at least three replicates. The standard deviations are indicated in the figures.
Randomization	There was no randomized experimental groups in this study.
Blinding	There was no grouping and blinding group applied in this study.

Reporting for specific materials, systems and methods

Materials & experimental systems

n/a	Involved in the study	
☒	☐	Unique biological materials
☐	☒	Antibodies
☐	☒	Eukaryotic cell lines
☒	☐	Palaeontology
☒	☐	Animals and other organisms
☒	☐	Human research participants

Methods

n/a	Involved in the study	
☒	☐	ChIP-seq
☒	☐	Flow cytometry
☒	☐	MRI-based neuroimaging

Antibodies

Antibodies used	Anti-HA antibody used in the immunoprecipitation experiment was purchased from Cell Signaling (catalog number: C29F4; lot number: 8). Anti-HA antibody used in the confocal microscopy analysis was purchased from SignalChem (catalog number: H98-63R; lot number: Q356-1). Anti-flag antibody was purchased from Sigma (catalog number: F1804; lot number: SLBS3530V). Anti-His antibody was purchased from CusAb (catalog number: CSB-MA000011M0m; lot number: D0612; clone number: 3G5). Alexa 488-conjucated anti-rabbit (catalog number: 111-545-003; lot number: 116143) and Cy5-conjugated anti-mouse (catalog number: 115-585-062; lot number: 122994) IgGs were purchased from Jackson ImmunoResearch Laboratories, Inc. The homemade rabbit anti-KDM8 antibody was raised against a full length of recombinant KDM8 protein.
Validation	The anti-HA, Flag, and His-tag antibodies specifically recognize the recombinant tags, respectively. They are applicable in Western blotting, immunoprecipitation, and immunofluorescence analysis as indicated in this study. The detailed specifications

of each primary antibody can be found in the manufacturers' websites.
Anti-HA (Cell Signaling): https://www.cellsignal.com/products/primary-antibodies/ha-tag-c29f4-rabbit-mab/3724
Anti-HA (SignalChem): https://shop.signalchem.com/products/anti-ha-h98-63r-25
Anti-Flag (Sigma): https://www.sigmaaldrich.com/catalog/product/sigma/f1804?lang=en&region=TW
Anti-His (CusAb): https://www.cusabio.com/Tag-Control-Antibodies/6*His-Monoclonal-Antibody-1028790.html
The homemade rabbit anti-KDM8 antibody had been validated in the previous publication (Wang et al., 2014, PNAS).

Eukaryotic cell lines

Policy information about cell lines

Cell line source(s)	The MCF-7 cell line was purchased from the Bioresource Collection and Research Center (BCRC) of Food Industry Research and Development Institute, Hsinchu, Taiwan.
Authentication	The MCF-7 cell line was purchased and authenticated by BRCR, Taiwan.
Mycoplasma contamination	All cell lines were confirmed to be free from Mycoplasma contamination.
Commonly misidentified lines (See ICLAC register)	No commonly misidentified cell line was used in this study.
